# Trained Circulating Monocytes in Atherosclerosis: *Ex Vivo* Model Approach

**DOI:** 10.3389/fphar.2019.00725

**Published:** 2019-06-27

**Authors:** Nikita G. Nikiforov, Reinhard Wetzker, Marina V. Kubekina, Anna V. Petukhova, Tatiana V. Kirichenko, Alexander N. Orekhov

**Affiliations:** ^1^National Medical Research Center of Cardiology, Institute of Experimental Cardiology, Moscow, Russia; ^2^Institute of Gene Biology, Centre of Collective Usage, Moscow, Russia; ^3^Institute of General Pathology and Pathophysiology, Moscow, Russia; ^4^Department of Anesthesiology and Intensive Care Medicine, Jena University Hospital, Jena, Germany; ^5^Institute of Human Morphology, Moscow, Russia

**Keywords:** atherosclerosis, monocyte, inflammation, activation, training, tolerance, intima, lipoproteins

## Abstract

Inflammation is one of the key processes in the pathogenesis of atherosclerosis. Numerous studies are focused on the local inflammatory processes associated with atherosclerotic plaque initiation and progression. However, changes in the activation state of circulating monocytes, the main components of the innate immunity, may precede the local events. In this article, we discuss tolerance, which results in decreased ability of monocytes to be activated by pathogens and other stimuli, and training, the ability of monocyte to potentiate the response to pathological stimuli, and their relation to atherosclerosis. We also present previously unpublished results of the experiments that our group performed with monocytes/macrophages isolated from atherosclerosis patients. Our data allow assuming the existence of relationship between the formation of monocyte training and the degree of atherosclerosis progression. The suppression of trained immunity *ex vivo* seems to be a perspective model for searching anti-atherogenic drugs.

## Innate Immunity and Atherosclerosis

Atherosclerosis remains a major problem of modern medicine, accounting for a substantial proportion of cardiovascular morbidity and mortality worldwide. Inflammation is one of the key mechanisms of atherosclerosis pathogenesis. Pro-inflammatory cells and molecules are present in atherosclerotic lesions and are currently regarded as potential therapeutic targets. According to current understanding, atherosclerotic lesion development can be regarded as a local chronic inflammatory process (Bentzon et al., 2014; Nikiforov et al., 2017a). At the cellular level, the likely triggering event in atherosclerosis is the accumulation of lipids, mainly cholesterol and its esters, in the cells constituting the intima-media layer of the arterial wall. The resulting foam cells owe their name to the abundant lipid inclusions filling their cytoplasm. The source of accumulating lipids is circulating modified low-density lipoprotein (LDL). Modified LDL particles are prone to formation of large self-associates, which are captured by macrophages. Previous studies by our group have identified the top 10 master regulator genes responsible for intracellular lipid accumulation by analyzing the transcriptome of macrophages that accumulated cholesterol as a result of modified LDL treatment (Orekhov et al., 2018b). The majority of the identified genes were associated with the immune response and inflammation. The obtained results were well correlated with the results reported by other authors, who showed that modified LDL initiated the secretion of pro-inflammatory molecules (Wiesner et al., 2010; Yang et al., 2014). One unexpected observation was that none of the identified master regulators were directly related to intracellular cholesterol metabolism. This suggested that the immune response may play a key role in foam cells formation.

Recent studies show that local inflammation in tissues can be preceded by earlier events taking place in the circulation (Edsfeldt et al., 2015) or even in the bone marrow (Libby and Ebert, 2018). Study of correlation between cytokines circulating in the blood of patients with atherosclerosis and the same cytokines in the atherosclerotic plaques yielded some interesting results. Concentrations of macrophage inflammatory protein-1b (MIP-1b), tumor necrosis factor (TNF), and fractalkine significantly correlated not only with the contents of the same cytokines in the plaques, but also with the contents of other pro-inflammatory molecules, such as interferon gamma (IFNγ), C-C motif ligand 2 (CCL2), and interleukin 6 (IL-6) (Edsfeldt et al., 2015). It is also known that monocytes isolated from the blood of atherosclerosis patients respond more strongly to lipopolysaccharide (LPS) stimulation, demonstrating increased expression and secretion of inflammatory markers. The facilitated activation of monocytes in patients with atherosclerosis may result from trained immunity (Bekkering et al., 2016).

Monocytes are key cells of the innate immunity present in the circulation that penetrate the arterial wall upon activation. Changes in monocyte inflammatory activation may hinder the resolution of inflammation in atherosclerotic lesions and contribute to the disease progression.

It is well known that atherosclerotic clinical manifestations are associated with increased monocyte activability. Mononuclear cells of unstable angina patients with recurrent phases of instability exhibit enhanced production of IL-6 in response to low-dose of LPS, which is correlated with baseline CRP levels (Liuzzo et al., 2001). The observed higher monocyte sensitivity seems to be a result of enhanced expression of TLR4 in circulating monocytes likewise detected in patients with unstable angina and acute myocardial infarction (Methe et al., 2005). At the same time, the association between CD14+/TLR-4+ monocytes of patients before cardiovascular events and future cardiovascular events was not detected (Lorenzen et al., 2011). These findings indicate that atherosclerotic clinical manifestations effect on CD14+/TLR-4+ monocytes contributing to enhanced pro-inflammatory response. However, the reasons of increased sensitivity of monocytes of atherosclerotic patients without clinical manifestations remain unclear.

## Tolerance and Training: Opposite Manifestations of the Innate Immune Memory

Tolerance of the innate immunity is one of the mechanisms of the resolution of inflammation. This phenomenon is characterized by the loss of sensitivity of monocytes/macrophages to repeated exposure to the pathogen (Dobrovolskaia and Vogel, 2002; Dobrovolskaia et al., 2003). This loss of sensitivity is manifested by reduced expression and secretion of the major pro-inflammatory cytokines and chemokines TNFα, IL-6, IL-1RA, CX3CR1, IL-10, HLA-DR, IL-8, CCL2, and IL-1, by monocytes/macrophages that become “tolerant” (Medvedev et al., 2006; Biswas and Lopez-Collazo, 2009). Tolerance is believed to be evolutionarily formed to protect body tissues from damage due to hyper-inflammation (Ifrim et al., 2014). However, tolerance can have negative consequences when the body is exposed to pathogens for a long time. LPS is the most studied inducer of tolerance. LPS is recognized by toll-like receptor 4 (TLR4), which triggers two signaling pathways. One of these pathways leads to the activation of IFN regulatory factor 3 (IRF3), while the other activates mitogen-activated protein kinases (MAPKs) and IkB kinase (IKK) complexes, which activate the transcription factors AP-1 and NF-kB, respectively. Together, IRF3, AP-1, and NF-kB transcription factors are responsible for the expression of inflammatory genes induced by LPS (Seeley and Ghosh, 2017).

Prolonged exposure to high doses of LPS provokes tolerance that develops as a result of the interaction of many factors involved in the transmission of signals from toll-like receptors including decreased expression of TLR4 itself, MyD88–TLR4 association, IL-1R-associated kinase (IRAK) activity, IkBa degradation (Medvedev et al., 2000; Nomura et al., 2000), and increased expression of some negative regulators, such as A20 and IRAK-M (Xiong et al., 2011), and some microRNAs that bind to different agents in the signal transmission chain or modulate their expression (Quinn et al., 2012). However, negative regulation of the TLR4 signaling pathway is not the only mechanism of tolerance formation. Recent studies have demonstrated that tolerance induction in macrophages is accompanied by chromatin remodeling, which blocks the access of transcription factors to a number of genes involved in TLR signal transduction (Foster et al., 2007). Other factors are also able to participate in the formation of tolerance, including nucleosome remodeling and DNA methylation and metabolic changes, which may contribute to the duration of the tolerance effect (Seeley and Ghosh, 2017).

Several studies have shown that tolerant macrophages have increased phagocytic activity, which in turn may play an important role in foam cells formation. However, in those studies, latex beads with immobilized components of the bacterial membrane were used to induce phagocytosis (Jing et al., 2013; de Lima et al., 2014).

TLRs are not the only regulators of tolerance formation. It was demonstrated that tolerance to TNFα is characterized by the loss of susceptibility of cells to re-stimulation with TNFα after a prolonged incubation of cells with this cytokine (Zwergal et al., 2006). Interestingly, a so-called “cross-tolerance” between TNFα and LPS has been observed, in which cells lost their sensitivity to TNFα after stimulation with LPS and *vice versa* (Park et al., 2011).

Recently, Netea with co-authors systematically investigated the role of pattern recognition receptors in the induction of long term responses of the innate immune system. It turned out that the interaction of cells with ligands that bind to NOD-like receptors (NOD2 receptors or NOD1 receptors), as well as to the dectin 1 receptor, induce a sensitization effect: repeated interaction of monocytes with the pathogen caused not a reduced, but increased pro-inflammatory cell activation, compared to the primary effect. Such phenomenon was called “training of innate immunity,” which is the exact opposite of tolerance (Ifrim et al., 2014). Interestingly, in some cases, low concentrations of TLR ligands (0.001–10 pg/ml of LPS) not only diminished tolerance, but also promoted training, thereby forcing monocytes to maintain the inflammatory status (Ifrim et al., 2014). Trained immunity can be caused by some pathogen-associated molecular patterns (PAMPs) and damage-associated molecular patterns (DAMPs), as well as oxidized LDL and Lp (a) (Munoz Villa, 1989; Kleinnijenhuis et al., 2012; Quintin et al., 2012; van der Valk et al., 2016).

Long-term tolerance responses of innate immune cells can be suppressed by both primary and repeated stimulation. Thus, interferons alpha and gamma are capable of such an effect by causing remodeling of the chromatin region responsible for tolerance formation (Bentzon et al., 2014; Shi et al., 2015). It can be concluded that some factors can modulate tolerance and training from one direction to another.

## Are Monocytes Trained in Atherosclerosis?

The inflammatory responses of circulating monocytes isolated from atherosclerotic patients have been evaluated in a clinical study that included healthy donors (N = 13) and patients with subclinical atherosclerosis (N = 23). Quantitative diagnostics of atherosclerotic states was performed by ultra-sonographic measurement of intima-media thickness (IMT) of common carotid arteries in high-resolution regimen. For this purpose, the distal portions of right and left carotid arteries were scanned in lateral angle of interrogation. IMT of common carotid arteries was measured on the far wall of the distal 10-mm segment before the area of carotid sinus. To assess the presence of atherosclerotic plaques, the examination also included a scan of the left and right common carotid arteries, the carotid sinus area, as well as external and internal carotid arteries in three fixed projections—anterior, lateral, and posterior. When visualizing atherosclerotic plaque, carotid arterial stenosis was assessed in transverse projection. The measurements of IMT and plaque stenosis were carried out with M’Ath computer software (Metris, SRL, France). The average of two measurements (from right and left arteries in lateral position) was considered an integral indicator of mean IMT. The following plaque score was used for analysis: 0—absence of plaque, 1—stenosis up to 20%, 2—stenosis 20–50%, 3—more 50% stenosis. Stenosis of the carotid artery lumen more than 20% was considered as defined atherosclerotic plaque. Other patient characteristics recorded in the study were age, gender, body mass index (BMI), Tchol, Tg, LDLc, HDLc, and statin usage (**Table 1**). The study protocol has been approved by the Institute for Atherosclerosis Research Committee on Human Research and meets the standards of the Declaration of Helsinki in its revised version of 1975 and its amendments of 1983, 1989, and 1996 (JAMA 1997;277:925–926). All study participants were free of cardiovascular disease. The extent of asymptomatic atherosclerosis was assessed using the data on IMT variability in apparently healthy individuals from the Russian population as described previously (Orekhov et al., 2015). Individuals belonging to the lowest and second quartiles of age-adjusted IMT distribution with no evidence of visible atherosclerotic plaques in any segment of carotid arteries were considered to be non-predisposed to atherosclerosis (“healthy”). Patients belonging to the third and fourth quartiles of IMT distribution with visible atherosclerotic plaques (more than 20% of the arterial lumen) in at least one segment of carotid arteries were considered having subclinical (asymptomatic) atherosclerosis. Patients belonging to the third and fourth quartile of IMT distribution with no atherosclerotic plaques visualized in any segment of carotid arteries were excluded from the study as intermediate. The sample size was sufficient to form statistically significantly different groups of subjects for IMT and monocyte activation.

**Table 1:** Baseline characteristics of the participants and pro-inflammatory response of circulating monocytes.

Characteristics	Healthy individuals (n = 13)	Subclinical atherosclerosis (n = 23)	p value
Age, y	63 ± 3	69 ± 2	0.09
Gender, % male (n)	8 (1)	30 (7)	0.08
BMI (kg/m2)	25.3 ± 1.1	28.7 ± 1.2	0.08
IMT	0.75 ± 0.03	0.90 ± 0.03	0.00**
Plaque score	0.6 ± 0.1	2 ± 0.1	0.00**
TChol, mmol/L	6.1 ± 0.3	5.8 ± 0.9	0.42
Tg, mmol/L	2.5 ± 1.3	1.2 ± 0.1	0.36
LDLc, mmol/L	3.9 ± 0.3	3.5 ± 0.2	0.34
HDLc, mmol/L	1.8 ± 0.1	1.7 ± 0.1	0.42
Statin use, % yes (n)	15 (2)	30 (7)	0.30
			
TNF expression in non-stimulated monocytes	0.010 ± 0.003	0.016 ± 0.002	0.16
TNF expression in LPS-stimulated monocytes	0.029 ± 0.004	0.052 ± 0.009	0.02*
TNF secretion by non-stimulated monocytes, pg/ml	689 ± 174	494 ± 55	0.30
TNF secretion by LPS-stimulated monocytes, pg/ml	3763 ± 332	4623 ± 317	0.05*

Data are presented as mean ± SD or n (%). BMI, body mass index; IMT, intima-media thickness; TChol, total cholesterol; Tg, triglycerides; LDLc, low density lipoprotein cholesterol; HDLc, high density lipoprotein cholesterol; TNF, tumor necrosis factor; LPS, lipopolysaccharide. Plaque score characterizes the degree of stenosis: 0—no stenosis, 1—less than 20%, 2—20–50%, 3—over 50%. CD14-positive monocytes were isolated from participants and incubated with or without LPS (1 mkg/ml) for 24 h. Then TNF secretion and expression were measured by ELISA and qPCR respectively.

*p < 0.05; **p < 0.01.

Monocytes were isolated from patients using magnetic CD14+ separation and incubated with 1 µg/ml of LPS for 24 h. After that, the secretion and expression levels of TNFα was measured using ELISA and qPCR respectively. The secretion and expression levels of TNFα were significantly increased in LPS-stimulated monocytes isolated from atherosclerotic patients compared with healthy participants (Table 1). It turned out that plasma TNF level was low and did not exceed 23 pg/ml as well as no significant difference between atherosclerotic patients and healthy individuals was observed. This finding relates well with different study of systematic mediators of inflammation in asymptomatic patients (Montecucco et al., 2010).

**Figure 1:**
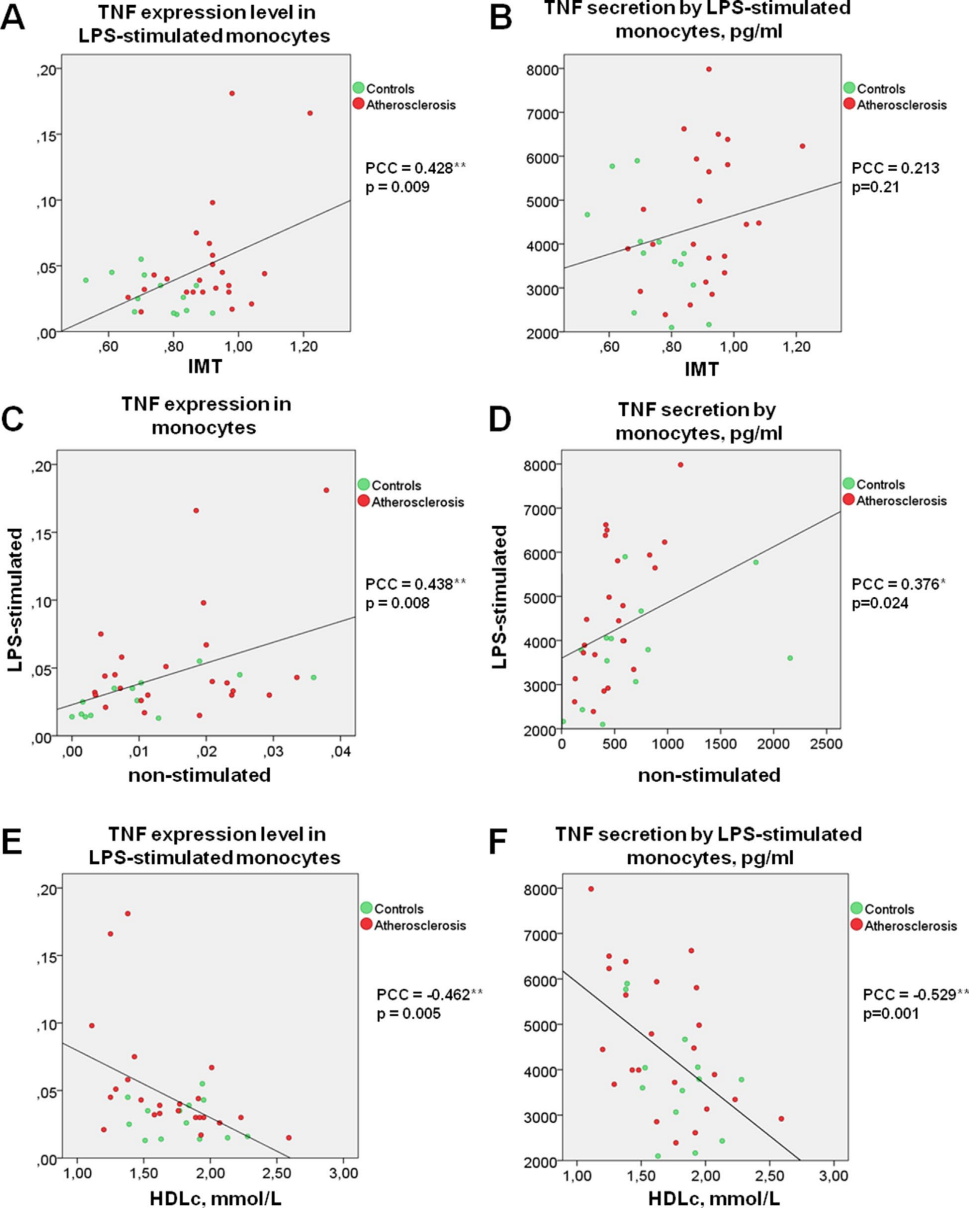
TNF secretion and expression level by human monocytes under LPS stimulation correlates directly with IMT and inversely with HDL cholesterol. **(A** and** B)** Correlation between TNF expression **(A)** or secretion **(B)** level by LPS-stimulated monocytes and IMT. **(C** and** D)** Correlation between the abilities of LPS-stimulated and non-stimulated monocytes express **(C)** or produce **(D)** TNF. **(E** and **F)** Correlation between TNF expression **(E)** or secretion **(F)** level by LPS-stimulated monocytes and HDL cholesterol. Each point on graphs corresponds to one patient. PPC, Pearson correlation coefficient.

Moreover, the significant correlation between TNFα expression by LPS-stimulated monocytes and IMT was observed (**Figure 1A**). Differences between the two groups of subjects correlated well with the results of another study (Patel et al., 2017). However, the key result of the current study was the observation that monocyte susceptibility to activation correlated not only with a discrete parameter (group number) but also with IMT. This may mean that there is a link between the formation of monocyte training and the degree of atherosclerosis progression. Previous studies by our group focused on the expression of TNFα in human lesions corresponding to different stages of atherosclerosis progression and found that it was maximal in lipofibrous plaques that are most enriched in lipids (Orekhov et al., 2018a). It is possible that the formation of monocyte tolerance with plaque thickening that may be responsible for the observed effect.

Interestingly, direct correlations of TNFα expression as well as secretion by non-stimulated and LPS-stimulated monocytes were observed for all participants (**Figure 1C, D**). It is therefore possible that the monocyte training effect is observed in response not only to TLR4 stimulation induced by LPS, but also to stressful conditions as a result of cell isolation, attachment, and cultivation.

## Monocyte Activatability and Lipid Profile

Surprisingly, secretion and expression of TNFα in LPS-stimulated monocytes demonstrated a strong negative correlation with participant’s HDL cholesterol (**Figure 1E, F**). Negative correlation between HDL cholesterol and TNFα secretion not only in LPS-stimulated, but also in non-stimulated monocytes (PPC = −0.692**, p <0.001) was observed. Our data suggest that the blood lipid profile may be an important factor determining the degree of the monocyte inflammatory response to a pathogen. At the same time, the lipid profile itself is a poor marker, since patients with atherosclerosis and healthy subjects did not significantly differ in Tchol, Tg, LDLc, and HDLc (**Table 1**). Furthermore, no significant correlation between HDLc and IMT was observed (PPC = −0.245, p = 0.162).

A recent report revealed a correlation between the lipid profile of healthy subjects and the ability of classical, non-classical, and intermediate monocyte subpopulations to respond to LPS stimulation (Patel et al., 2017). This study evaluated only intracellular cytokine production. Interestingly, an inverse correlation between HDLc and intracellular production of IL-1β was found. No significant correlation between the intracellular production of TNFα and Tchol, LDLc or HDLc was observed. Large individual differences in monocyte activation were also found. Unfortunately, patients with atherosclerosis did not participate in the study.

It is well known that HDL may have a protective, anti-inflammatory effect on the endothelial cells. However, its effect on monocytes and macrophages is less studied. Murphy et al. (2008) demonstrated that HDL caused a dose-dependent decrease in CD11b activation under the influence of PMA, and apolipoprotein A-I was responsible for the effect. ApoA-I acted *via* ABCA1, whereas HDL acted through several receptors. The ability of HDL to modulate the expression of inflammatory genes has also been studied (Colin et al., 2014). On the one hand, Colin S et al. showed that HDL had no effect on the formation of the alternatively-activated M2 phenotype of macrophages, which suggests that the anti-inflammatory properties of HDL do not manifest themselves through the enhancement of the anti-inflammatory phenotype M2. Finally, Lee et al. (2016) investigated the ability of HDL to modulate the differentiation of monocytes to pro-inflammatory M1 macrophages in the presence of LPS and IFNγ. It turned out that HDL reduced the expression of M1 macrophage surface markers CD192 and CD64, as well as pro-inflammatory genes TNFα, IL-6, and MCP-1 (CCL2). The authors demonstrated that reverse cholesterol transport played an important role in the observed effect.

Recently, we analyzed the transcriptome of HDL-treated monocyte-derived macrophages in order to identify genes that could be upregulated by HDL (Orekhov et al., 2018c). Only three identified genes were significantly up-regulated by HDL-treatment: fatty acid desaturase 1 (FADS1, regulates unsaturation of fatty acids), insulin induced gene 1 (INSIG1, regulates lipid synthesis), and the low-density lipoprotein receptor (LDLR, binds non-modified LDL). In parallel, the role of identified genes in cholesterol efflux was investigated. We found that knockdown of INSIG1 and LDLR using siRNA decreased cholesterol efflux down-regulating the expression of ABCA1 and ABCG1. Thus, HDL particles seem to activate genes involved in lipid biosynthesis and these genes are required for successful cholesterol efflux. FADS1, INSIG1, and LDLR are regulated through SREBP2 pathway likely as a result of the reduction of the cellular cholesterol content. HDL particles may also affect on basic mediators regulating not only lipid biosynthesis but other anabolic processes (Nagao et al., 2017) as well as maintenance and repair reactions (Kimura et al., 2010). Target of rapamycin (mTOR), central mediator of anabolic processes including activation of SREBPs with further lipid biosynthesis, is involved in the induction of trained immunity (Cheng et al., 2014). It is assumed that AMP activated protein kinase (AMPK) plays a critical role in development of immune tolerance (Kim et al., 2014). Thus, we can speculate that HDL particles accelerate cholesterol efflux and lipid biosynthesis affecting on monocyte sensitivity *via* energy mediators (Bauer et al., 2018).

## Suppression of Trained Immunity *Ex Vivo* as a Model for Searching Anti-Atherogenic Drugs

It appears therefore that trained immunity is an unfavorable phenomenon that can contribute to chronic inflammation. Suitable models are needed to develop approaches to reducing the monocytes reactivity. Previous works by our group used an *in vitro* model to identify the anti-atherogenic and anti-inflammatory properties of various pharmacological agents (Nikiforov et al., 2017b). However, this approach gave no results. The *ex vivo* model turned out to be much more effective. In this model, patients with atherosclerosis were given an investigational drug, and blood samples were collected at 0, 2, and 4 h. Monocytes were isolated from blood samples taken before the drug administration (0 hours) and after 2 and 4 h. After that, monocytes were cultivated in the presence of a pro-inflammatory activator (most often IFNγ, less often LPS) for 24 h, after which the TNFα expression in stimulated and non-stimulated cells was measured. In parallel, serum was isolated from blood samples, which were tested for their ability to induce the accumulation of cholesterol in cultured control monocytes isolated from the blood of healthy donors (Nikiforov et al., 2017c). Usually, blood serum of patients with atherosclerosis is atherogenic, i.e., causes the accumulation of cholesterol in cultured monocytes when added at 10% for 24 h. Blood serum of healthy donors, which did not exhibit atherogenic effect, was used as a control (Orekhov et al., 2014).

The described model was used to evaluate four medicinal products: Allicor (INAT-Farma, Russia), CardioHealth (Sweden), Cellex (Pharm-Sintez, Russia), and Vezugen (JSC “Pharm,” Russia). It turned out that Allicor and CardioHealth caused a significant decrease of TNFα expression in non-stimulated monocytes isolated from blood taken 4 h after drug administration and a tendency to suppression of response by stimulated cells. Four hours after receiving Allicor or CardioHealth, a significant decrease in the ability of blood serum to cause cholesterol accumulation in a primary macrophage culture was observed as well.

The observed increased pro-inflammatory sensitivity of monocytes from atherosclerotic patients and its correlation with IMT allowed us to assume that trained monocytes can make a significant contribution to chronic inflammation development in the arterial wall. Thus, trained phenotype of circulating monocytes can be considered as a perspective target for anti-atherosclerotic therapy. This *ex vivo* model seems well suited for identifying drugs that have an ability to reduce the reactivity of circulating monocytes and, as a consequence, be of interest for the development of anti-atherosclerotic immune-corrective therapy.

## Concluding Remarks

In conclusion, the most significant factor associated with IMT appears to be the level of TNFα expression in monocytes. Interestingly, there was a correlation between HDLc and TNFα secretion not only in LPS-stimulated but also in non-stimulated monocytes, which in turn may indicate tolerance induction by HDL independent on TLR4 stimulation. Possibly, stressful conditions caused by the procedure of isolating monocytes and their attachment in culture expressed ability to sensitize monocytes provoking long-term training responses. Of course, this interpretation is still rather speculative, and the mechanism of HDL’s influence on the training remains to be elaborated.

It is likely that, due to the ability of HDL to produce cholesterol outflow from circulating cells directly into the bloodstream, there may be a correlation between cholesterol (and cholesterol esters) in circulating monocytes and their ability to be activated. It may turn out that in the logical chain “low HDLc—trained monocytes—high IMT” one link is missing. This link might be directly or indirectly related with the intracellular cholesterol content in circulating monocytes.

## Ethics Statement

This study was carried out in accordance with the recommendations of Institute for Atherosclerosis Research Committee on Human Research and meets the standards of the Declaration of Helsinki in its revised version of 1975 and its amendments of 1983, 1989, and 1996 (JAMA 1997;277:925–926) with written informed consent from all subjects. All subjects gave written informed consent in accordance with the Declaration of Helsinki. The protocol was approved by the Atherosclerosis Research Committee on Human Research.

## Author Contributions

NN conceptualized, performed experimental studies, literature search, and supervision of the research project, and wrote the manuscript. TK conducted the patients’ recruitment, clinical examination, and clinical data acquisition. Cell culture experiments were performed by AP and MK. Manuscript editing was done by RW and AO.

## Funding

This work was supported by the Russian Foundation for Basic Research (Grant No. 18-34-00997) for the genetic and cell culture experiments and by the Russian Science Foundation (Grant No. 19-15-00010) for the studies on patients.

## Conflict of Interest Statement

The authors declare that the research was conducted in the absence of any commercial or financial relationships that could be construed as a potential conflict of interest.
